# Leveraging Clinical Record Geolocation for Improved Alzheimer’s Disease Diagnosis Using DMV Framework

**DOI:** 10.3390/biomedicines13102496

**Published:** 2025-10-14

**Authors:** Peng Zhang, Divya Chaudhary

**Affiliations:** Khoury College of Computer Sciences, Northeastern University, Seattle, WA 98109, USA

**Keywords:** Alzheimer’s disease, machine learning, large language models

## Abstract

**Background:** Early detection of Alzheimer’s disease (AD) is critical for timely intervention, but clinical assessments and neuroimaging are often costly and resource intensive. Natural language processing (NLP) of clinical records offers a scalable alternative, and integrating geolocation may capture complementary environmental risk signals. **Methods:** We propose the DMV (Data processing, Model training, Validation) framework that frames early AD detection as a regression task predicting a continuous risk score (“data_value”) from clinical text and structured features. We evaluated embeddings from Llama3-70B, GPT-4o (via text-embedding-ada-002), and GPT-5 (text-embedding-3-large) combined with a Random Forest regressor on a CDC-derived dataset (≈284 k records). Models were trained and assessed using 10-fold cross-validation. Performance metrics included Mean Squared Error (MSE), Mean Absolute Error (MAE), and R^2^; paired t-tests and Wilcoxon signed-rank tests assessed statistical significance. **Results:** Including geolocation (latitude and longitude) consistently improved performance across models. For the Random Forest baseline, MSE decreased by 48.6% when geolocation was added. Embedding-based models showed larger gains; GPT-5 with geolocation achieved the best results (MSE = 14.0339, MAE = 2.3715, R^2^ = 0.9783), and the reduction in error from adding geolocation was statistically significant (*p* < 0.001, paired tests). **Conclusions:** Combining high-quality text embeddings with patient geolocation yields substantial and statistically significant improvements in AD risk estimation. Incorporating spatial context alongside clinical text may help clinicians account for environmental and regional risk factors and improve early detection in scalable, data-driven workflows.

## 1. Introduction

Alzheimer’s disease is a progressive neurodegenerative disorder that affects millions of individuals worldwide [1]. Characterized by cognitive decline, memory loss, and behavioral changes, Alzheimer’s is the most common cause of dementia among older adults. As the global population ages, the prevalence of Alzheimer’s disease is expected to rise significantly, posing a substantial burden on healthcare systems and families [2,3]. Early detection of Alzheimer’s is crucial for managing symptoms, delaying progression, and improving the quality of life for affected individuals [4]. However, the subtle onset of the disease often leads to delayed diagnosis, limiting the effectiveness of interventions [5]. Recent advancements in medical technology have emphasized the importance of early detection of AD. Traditional methods rely heavily on clinical assessments and neuroimaging, which can be time-consuming and expensive. The integration of Natural Language Processing (NLP) into medical diagnostics offers a promising alternative, allowing for the analysis of patient records to identify early signs of cognitive decline. Recent advancements in NLP allow us to extract meaningful patterns from such unstructured text data, potentially enhancing our ability to predict early AD onset [6].

Despite the potential of NLP in medical diagnostics, there remains a gap in leveraging geolocation data alongside textual analysis for early detection of Alzheimer’s disease. While [7] shows that patient records contain valuable information about cognitive function, the incorporation of spatial data, such as geolocation, has been largely unexplored in this context. Geolocation data, which includes information about an individual’s physical movements and locations, could provide additional insights into behavioral patterns that correlate with the early stages of Alzheimer’s [8]. The problem this research addresses is the lack of integrated approaches that combine geolocation data with advanced NLP models to enhance the early detection of Alzheimer’s disease.

The primary contributions of this paper are threefold. First, we introduce a regression-based DMV framework for early AD detection, focusing on predicting a continuous AD risk score rather than discrete categories. Second, we utilize Llama3-70B, GPT-4o, and GPT-5 language models, which capture contextual and linguistic subtleties that may be missed by traditional NLP models like BERT. Third, we incorporate geolocation data as an additional feature, demonstrating its impact through ablation studies that evaluate the model’s sensitivity to different feature subsets. Our experimental results indicate that this approach significantly improves the accuracy of early AD risk predictions, providing valuable insights for clinicians in neurology and geriatrics. This framework will enable the models to be trained on a broader range of data, potentially improving their generalizability and robustness across different demographics and clinical settings [9,10].

The remainder of this paper is organized as follows. Section 2 reviews related work on AD detection using NLP and discusses the advantages of regression-based modeling. Section 3 describes the dataset, detailing the clinical notes and geolocation features. Section 4 outlines our DMV framework, including data processing, the model architecture, and validation. Section 5 presents experimental results, including ablation studies, performance comparisons, and statistical tests, and then discusses the clinical implications of our findings and concludes the paper.

## 2. Related Work

The application of Natural Language Processing (NLP) and machine learning techniques in the early detection of Alzheimer’s disease has garnered significant attention in recent years. Various studies have explored the use of NLP to analyze patient records, clinical notes, and other text-based data to identify early signs of cognitive decline.

### 2.1. Foundational Works

One of the foundational works in this area is by [11], who developed an NLP-based approach to identify Alzheimer’s disease from clinical notes. By employing a combination of named entity recognition and sentiment analysis, the study successfully identified early indicators of Alzheimer’s, such as memory loss and confusion, in patient records. Similarly, Ref. [12] utilized NLP to analyze speech and language patterns in transcribed conversations, demonstrating that certain linguistic features, such as reduced vocabulary diversity and increased usage of filler words, are correlated with the early stages of Alzheimer’s.

In addition to NLP, machine learning models have been widely applied to the problem of Alzheimer’s detection. Ref. [13] leveraged support vector machines (SVMs) and Random Forests to classify patients based on cognitive assessments and clinical data. Their study emphasized the importance of feature selection and data preprocessing in improving model accuracy. Another noteworthy contribution is by [14], who implemented deep learning techniques, including convolutional neural networks (CNNs) to analyze medical images and text data simultaneously. Their multimodal approach showed improved diagnostic accuracy compared to single-modality models.

The introduction of transformer-based models, such as Llama3, GPT-4o, and GPT-5, has further advanced the capabilities of NLP in medical applications. Ref. [15] introduced Llama3, which quickly became a standard in NLP due to its ability to capture complex linguistic features and contextual subtleties. Llama3 has been applied to various medical NLP tasks, including the extraction of disease-related information in the radiology field [16]. Ref. [17] presented GPT-4 which demonstrated significant advancements in generating coherent and contextually relevant text; later iterations such as GPT-4o and GPT-5 have continued to improve representation quality for clinical language. The potential of these models for early Alzheimer’s detection has been explored in preliminary studies, showing promise in identifying subtle linguistic cues that are associated with cognitive decline.

#### Survey-Based AD Risk Assessment

Population-level surveys have become an important tool for AD risk assessment and early detection. The CDC’s BRFSS system, which our dataset is derived from, represents the largest continuously conducted health survey in the world, with demonstrated reliability and validity across multiple health outcomes [18]. The Cognitive Decline Module specifically has been used to identify subjective cognitive decline (SCD), which research has established as a preclinical indicator of Alzheimer’s disease [19]. Multiple validation studies have shown that questionnaire-based SCD assessments significantly predict conversion to mild cognitive impairment and dementia, with hazard ratios ranging from 2.0 to 4.5 depending on the severity and persistence of reported cognitive concerns [20,21,22]. These findings support the use of survey responses as continuous risk indicators in predictive modeling frameworks.

### 2.2. Existing Gaps

While substantial progress has been made in utilizing NLP and machine learning for Alzheimer’s detection, several gaps remain that this research aims to address.

Although both Llama3 and GPT-4o have been applied to medical NLP tasks, there is limited research directly comparing their effectiveness in the context of Alzheimer’s detection. Most studies have focused on one model at a time, without a comprehensive comparison of their performance on similar datasets. This research seeks to fill this gap by evaluating the strengths and weaknesses of Llama3 and GPT-4o in predicting AD risk scores from patient records. Recent studies, such as [23,24], have begun to explore these comparisons in other medical applications, but specific studies focusing on Alzheimer’s detection remain scarce.

The potential of geolocation data in enhancing Alzheimer’s detection has been largely unexplored. Existing research has primarily focused on text-based analysis, neglecting the spatial and behavioral information that geolocation data can provide. By integrating geolocation data with NLP models, this research aims to uncover new patterns that could indicate the onset of Alzheimer’s disease, thereby providing a more holistic approach to early detection. Preliminary studies by [25] have shown promising results in other domains, suggesting that geolocation data can be a valuable addition to patient records for disease prediction.

The significance of geographic location in Alzheimer’s disease risk extends beyond simple demographic clustering. Environmental neurotoxins, particularly air pollutants like PM2.5 and nitrogen dioxide, have been linked to increased AD incidence through multiple epidemiological studies [26,27]. Access to healthcare resources varies dramatically by location, affecting both early detection rates and disease management quality. Socioeconomic factors tied to geography, including education levels, dietary patterns, and physical activity opportunities, create distinct risk profiles across regions.

For example, rural populations often face delayed diagnosis due to limited access to specialists, while urban areas may expose residents to higher pollution but provide better healthcare infrastructure. Regional variations in social support networks, community engagement opportunities, and cognitive stimulation resources all contribute to AD risk trajectories. By incorporating these location-based factors, our model can capture environmental and social determinants of health that traditional clinical assessments might overlook.

While many studies have shown promising results in controlled environments, there is a need for research that validates these approaches in real-world settings. The integration of advanced NLP models with geolocation data and their application to diverse patient populations could lead to more robust and generalizable diagnostic tools. Refs. [28,29] have highlighted the challenges in translating AI models from research to real-world applications, emphasizing the importance of validating these models across diverse settings.

By addressing these gaps, this research contributes to the ongoing efforts to improve early detection of Alzheimer’s disease, offering a novel approach that combines advanced NLP models with geospatial analysis.

## 3. Methodology

### 3.1. Data Description

The dataset used in this study is derived from the Centers for Disease Control and Prevention (CDC) and includes 284K records with 31 columns that contain a mix of categorical, numerical, and geolocation features with the latest updates on 29 April 2024. The dataset consists of patient records, clinical notes, cognitive assessments, and geolocation data that provide a comprehensive view of patient behavior and health status over time. The use of survey questions to assess AD risk and cognitive decline is well-established in public health research. The BRFSS Cognitive Decline Module has been validated across multiple studies as an effective tool for identifying individuals who are at risk of dementia [18,30].

**Figure 1:**
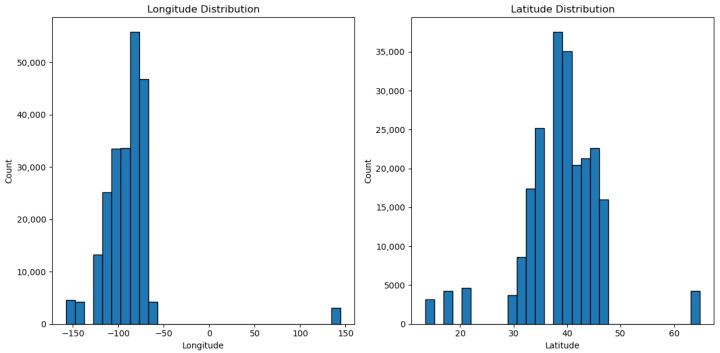
Distribution of Geolocation Features.

In our research, one of the key features is the patients’ locations, which are represented by latitude and longitude. We extract them from the “Geolocation” column. Figure 1 illustrates the distribution of both features in the dataset, which are latitude and longitude. The diagram shows that most patient records are from North America’s West Coast.

In addition, the dataset contains questions under 39 topics and corresponding values (data_value) for each question. Our target is the “data_value” in the dataset, which is the risk indicator, with higher value representing high risk of AD. Some sample questions and values are listed in Table 1. The scores represent the risk or possibility of developing Alzheimer’s disease for the patients in this area. The higher the score, the more likely the patients in this area are to have Alzheimer’s disease.

### 3.2. DMV Framework

**Figure 2:**
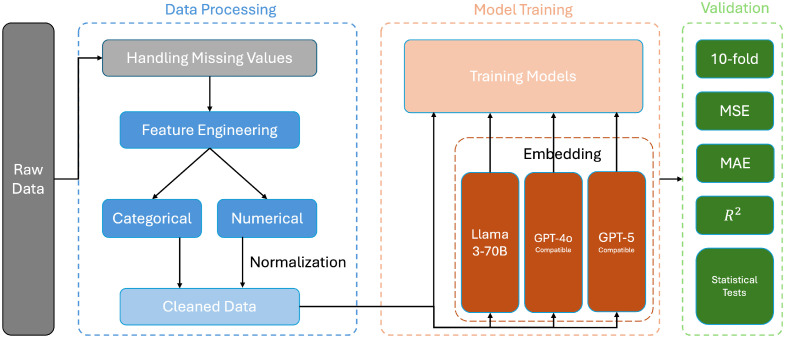
DMV framework visualization. The pipeline is split into three stages: Data Processing (cleaning, feature engineering and extraction/normalization of latitude/longitude), Model Training (text embeddings from Llama3-70B, GPT-4o- and GPT-5-compatible models fused with structured features and used to train regressors), and Validation (10-fold cross-validation reporting MSE, MAE and $R^{2}$, plus inferential tests to verify the statistical significance of geolocation).

We have built a framework to predict the Alzheimer’s disease (AD) risk score, which we have named the DMV framework (Data processing, Model training, and Validation). This acronym reflects the three core components of our methodology. It contains three main parts, data processing, model training, and validation, as shown in Figure 2. This robust framework ensures that it combines all useful features, including categorical and numerical features, to enable the model to see the data from a more comprehensive perspective and reach high accuracy with low error.

### 3.3. Data Preprocessing

**Figure 3:**
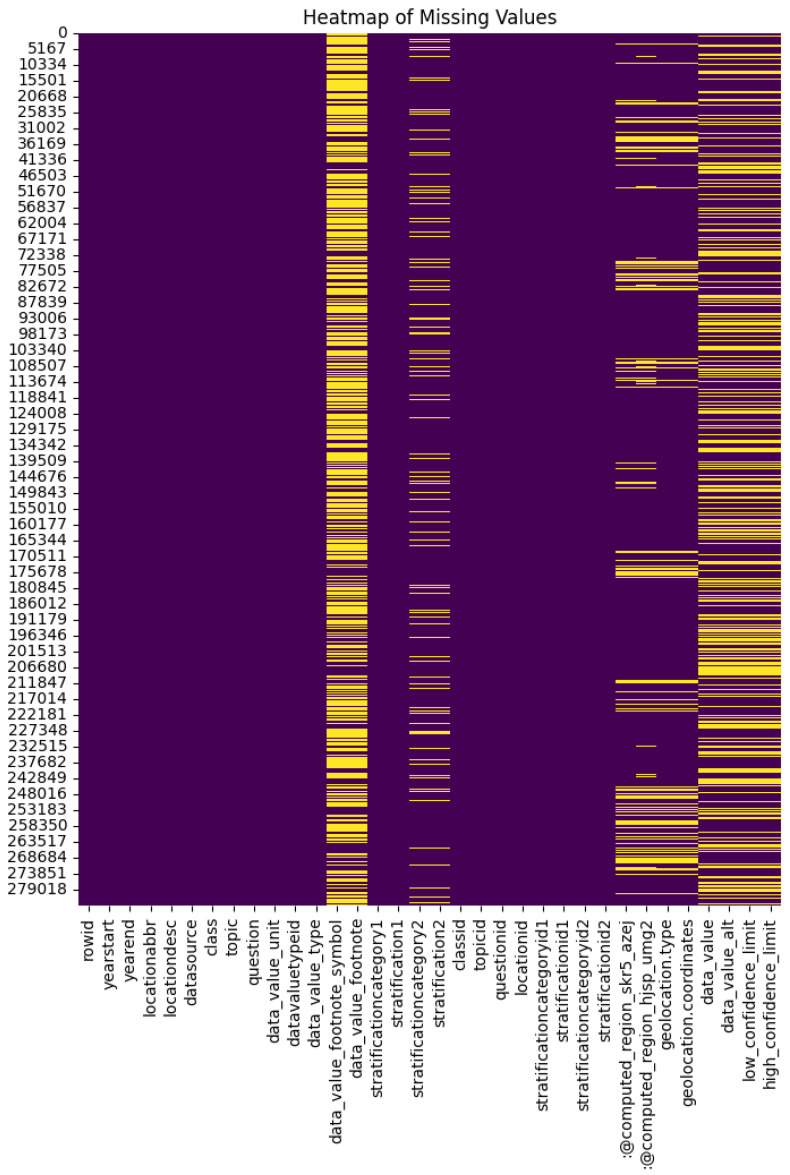
Heatmap of missing values across dataset features. Yellow indicates missingness.

Before model training, extensive data preprocessing steps were undertaken to ensure the dataset’s quality and usability:

**Handling Missing Values.** Missing values shown as yellow lines in Figure 3 were identified and addressed through imputation methods. For numerical columns, missing values were filled using the mean or median, while for categorical columns, the mode or a separate category labeled ‘Unknown’ was used [31].

**Feature Selection.** Feature selection was conducted to identify the most relevant columns for the task at hand. The original dataset contained 34 columns, of which 12 were excluded due to excessive missing values. We further removed six highly correlated columns (classid, topicid, questionid, locationid, rowid, and datavaluetypeid) as these served only as database keys without predictive value. For instance, the dataset includes both a ‘question’ column and a ‘questionid’ column, which have a one-to-one relationship. In this case, we chose to drop the ‘questionid’ column. The data_value_unit column was also excluded as it does not contribute to the prediction. After this preprocessing, 16 features remained for model development. With the remaining features, we split them into categorical and numerical groups as shown in Table 2 and Table 3 because we cannot feed them into models directly.

**Geolocation Extraction.** The raw dataset contained geolocation data in point-type format, encompassing latitude and longitude coordinates. We extracted these coordinates and stored them as two separate numerical columns (“latitude” and “longitude”) to enable their use as features in downstream experiments. These geospatial features were subsequently normalized along with other numerical variables.

**Encoding Categorical Data.** Categorical columns were transformed using One-Hot Encoding, ensuring that all categorical data were represented in a format that was suitable for model input [32].

**Normalization.** Numerical features were normalized to have a standard range, preventing bias toward any particular feature due to differences in scale [33].

### 3.4. Baseline

The baseline model employed in this study is the Random Forest Regressor (RF), a widely used ensemble learning method that operates by constructing a multitude of decision trees. It provides robust results by averaging the predictions of individual trees to mitigate overfitting [34].

### 3.5. Model Embeddings

For the advanced models, Llama3-70B, GPT-4o, and GPT-5 embeddings were leveraged to capture the linguistic nuances in patient records.

**Llama3-70B Embeddings.** Text data from patient records were tokenized and fed into the pre-trained Llama3-70B model. Following standard practices for extracting contextualized embeddings from transformer-based models [35,36], we extracted embeddings from the final hidden layer of the model. Specifically, the output of the last transformer layer (layer 80 for Llama3-70B) was used to generate contextualized representations for each token, which were then aggregated to produce document-level embeddings [37]. These embeddings represent the semantic meaning of the text and were used as features for the subsequent machine learning models. The embeddings were combined with other numerical and categorical features. The Random Forest Regressor was then trained on this enriched feature set, aiming to improve predictive accuracy.

**GPT-4o Compatible Embeddings.** For generating embeddings that are compatible with GPT-4o-based systems, we utilized OpenAI’s text-embedding-ada-002 model [38], which provides high-quality text representations with 1536 dimensions. The text-embedding-ada-002 model represents OpenAI’s second-generation embedding approach, unifying capabilities across text search, similarity, and code understanding tasks while achieving strong performance across diverse benchmarks [38]. Patient text records were processed through this embedding model to generate fixed-length vector representations. The process mirrored that of Llama3-70B, where embeddings were concatenated with other features and used to train the Random Forest Regressor. For ease of reference, we refer to embeddings generated using OpenAI’s text-embedding-ada-002 model as “GPT-4o embeddings” throughout this paper.

**GPT-5 Compatible Embeddings.** For GPT-5-compatible embeddings, we employed OpenAI’s text-embedding-3-large model, which produces embeddings with up to 3072 dimensions and represents a substantial improvement over previous generation models [39]. The text-embedding-3-large model achieved significant performance gains on multilingual retrieval benchmarks (MIRACL) and English task benchmarks (MTEBs) compared to text-embedding-ada-002 [39]. Text data were processed to produce these embeddings, which were concatenated with other numerical and categorical features and used to train the Random Forest Regressor. In our experiments, GPT-5-compatible embeddings produced the most informative representations and led to the best downstream performance. Similarly, we refer to embeddings generated using OpenAI’s text-embedding-3-large model as “GPT-5 embeddings”.

### 3.6. Robust Validation

To rigorously evaluate the performance of the DMV framework, we employed a 10-fold cross-validation approach. The model’s predictive accuracy was assessed using several key metrics: Mean Squared Error (MSE), Mean Absolute Error (MAE), and R-squared (R^2^). Additionally, we evaluated the impact of incorporating geolocation features on the model’s performance. By training and validating the DMV framework both with and without these location-based variables, we were able to assess whether the inclusion of spatial information improved the overall predictive accuracy.

### 3.7. Model Interpretability

Our DMV framework achieves interpretability through its structured feature engineering approach, which separates inputs into meaningful categorical and numerical components. The categorical embeddings capture clinical questions from the dataset that assess AD risk from multiple dimensions, including cognitive function, daily activities, mental health status, and physical health indicators. Each question corresponds to a specific aspect of AD risk assessment, as shown in Table 1, making it transparent which health dimensions contribute to the final risk score.

The numerical features, particularly the geolocation coordinates (latitude and longitude), provide an additional interpretable layer by capturing environmental and regional risk factors. Rather than operating as a black box, this separation allows clinicians to understand that location-based adjustments to risk scores reflect documented geographic variations in AD prevalence, environmental exposures, and healthcare access patterns discussed in Section 2.

## 4. Performances and Results

### 4.1. Model Performance

The performance of the models was evaluated using MSE, MAE, and R^2^ score. The results are summarized in Table 4.

**Baseline (RF).** The Random Forest Regressor (with geolocation) achieved an R^2^ of 0.9588 with an MSE = 20.1164 and an MAE = 2.9352, indicating that it explains about 95.9% of the variance in the target variable when spatial features are included.

**Llama3-70B + RandomForestRegressor.** Incorporating Llama3-70B embeddings (with geolocation) yielded an MSE = 16.6892, an MAE = 2.8776 and an R^2^ = 0.9695, improving over the baseline and demonstrating that semantic embeddings help the downstream regressor.

**GPT-4o + RandomForestRegressor.** GPT-4o (with geolocation) produced an MSE = 16.6118, an MAE = 2.8833 and an R^2^ = 0.9696, comparable to Llama3-70B on these metrics.

**GPT-5 + RandomForestRegressor.** GPT-5 (with geolocation) achieved the best overall results, an MSE = 14.0339, an MAE = 2.3715 and an R^2^ = 0.9783, indicating the strongest fit and lowest prediction error among the evaluated models.

Beyond quantitative metrics, the embedding models exhibited distinct strengths when processing clinical narratives. Llama3-70B was particularly effective at capturing medical terminology and maintaining consistency across longer clinical notes, likely due to domain-focused pretraining. GPT-4o and GPT-5 excelled in the contextual understanding of complex, multi-condition cases where AD symptoms interact with comorbidities; GPT-5 in particular, provided the most consistent and accurate representations in our experiments. GPT-4o sometimes over-interpreted ambiguous statements in sparse documentation, whereas GPT-5 produced more stable predictions across such cases.

For practical deployment, Llama3-70B may be preferred where highly standardized clinical documentation is the norm, while GPT-5 is recommended when diverse, unstructured input and the highest predictive accuracy are desired. GPT-4o remains a strong alternative when latency or cost constraints affect model selection.

In summary, the embedding-based approaches demonstrated superior performance compared to the baseline (RF). GPT-5 achieved the lowest MSE and MAE and the highest R^2^, while Llama3-70B and GPT-4o also provided substantial improvements over the baseline. These results validate the effectiveness of incorporating language model embeddings for improving prediction accuracy.

### 4.2. Cross-Validation and Runtime Summary

We used 10-fold cross-validation for the final evaluation and reported the mean and standard deviation of R^2^ across folds. Table 5 summarizes these results. Note that Table 4 reports the metrics from the final aggregated evaluation, whereas Table 5 reports the mean and standard deviation across the 10 cross-validation folds. Differences between the two tables reflect the different evaluation protocols (single aggregated run vs. CV means) and natural variability due to data splits.

### 4.3. Residual Analysis

**Figure 4:**
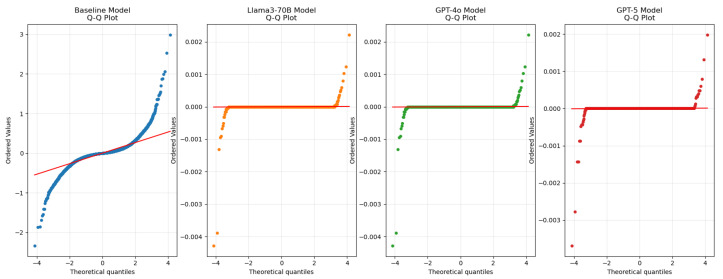
Q–Q plot of residuals for baseline (RF) and embedding-based models (Llama3-70B, GPT-4o, GPT-5). Points closer to the diagonal indicate better agreement with the normal distribution.

Figure 4 presents the updated Q–Q (quantile–quantile) plot for the residuals of the evaluated models. The Q–Q plot shows how closely residuals follow a normal distribution: points lying near the diagonal indicate good agreement. The embedding-based approaches (Llama3-70B, GPT-4o, and GPT-5) exhibit tighter adherence to the diagonal than the baseline RF, with GPT-5 showing the closest fit and the smallest deviations. These observations indicate that the embedding models, especially GPT-5, produce more consistent residuals and that there is no strong systematic bias in the predictions [40].

### 4.4. Feature Importance

Analysis of feature importance showed that textual embeddings are the primary driver of predictive performance, and that adding geolocation features consistently improves results across models. The observed metric changes (with vs. without geolocation) are summarized in Table 6, Table 7 and Table 8.

**Figure 5:**
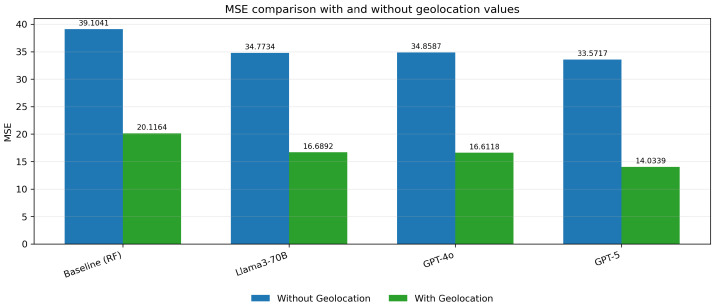
MSE comparison with and without geolocation values.

**Figure 6:**
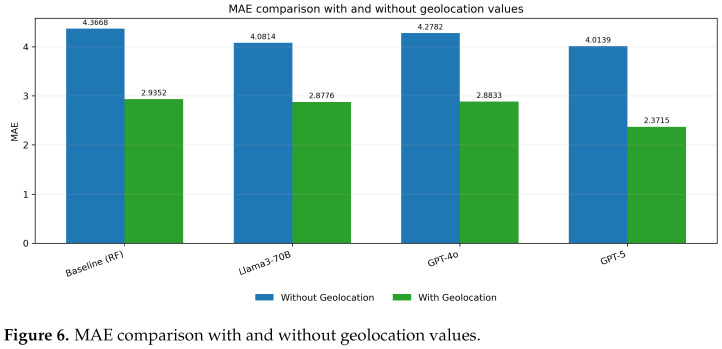
MAE comparison with and without geolocation values.

**Figure 7:**
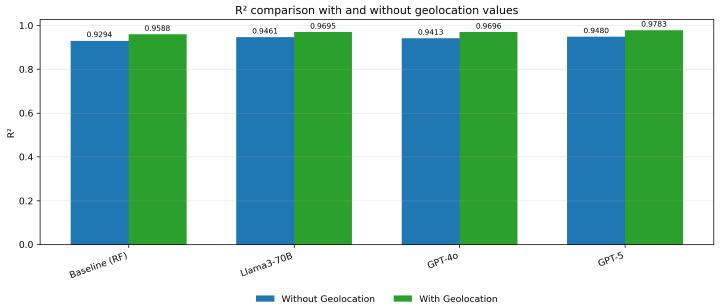
R^2^ comparison with and without geolocation values.

The MSE values for the embedding models (Llama3-70B, GPT-4o, and GPT-5) are significantly lower when geolocation data is included, indicating a reduction in prediction error across all embedding methods (Figure 5). Similarly, the MAE values are notably smaller with geolocation, reflecting improved accuracy in predictions. (Figure 6) The R^2^ values show an increase, demonstrating a better fit of the models to the data when geolocation is used (Figure 7).

### 4.5. Statistical Tests

To quantify whether the observed improvements from including geolocation features are statistically robust, we performed a set of inferential tests on the per-fold evaluation metrics (10-fold cross-validation results). The following procedures were applied:

- Paired two-sided Student’s t-tests comparing per-fold MSE values for each model with and without geolocation (significance threshold α=0.05).
- One-way ANOVA to assess differences among the set of models on per-fold MSE.
- Effect-size analysis using Cohen’s *d* to measure practical significance of the observed changes.
- Wilcoxon signed-rank tests as a non-parametric confirmation of the paired comparisons.

The test results are summarized below.

#### 4.5.1. Paired *t*-Tests (With Geo vs. Without Geo)

All paired *t*-tests were highly significant (*p* < 0.001), indicating that adding geolocation yields statistically detectable reductions in MSE on the per-fold evaluation vectors in Table 9.

#### 4.5.2. One-Way ANOVA

An omnibus one-way ANOVA across models returned F = 955.2452 (*p* < 0.001), supporting the presence of statistically significant differences among the evaluated models on per-fold MSE.

#### 4.5.3. Wilcoxon Signed-Rank Tests (Non-Parametric)

To verify that results are not dependent on the normality assumption, we ran Wilcoxon signed-rank tests on the per-fold MSE differences. These non-parametric tests corroborated the *t*-test findings (all *p* < 0.001) in Table 10.

##### Interpretation and Limitations

The inferential results indicate that including geolocation features leads to statistically significant reductions in MSE across all models, with GPT-5 showing the largest relative benefit. Therefore, our experiments suggest meaningful and practical gains from geolocation. While the statistical significance is consistent across parametric (*t*-tests) and non-parametric (Wilcoxon signed-rank) tests, the effect sizes are derived from a single cross-validation scheme and may not generalize to different geographic regions or time periods. Additionally, these findings should be interpreted with caution. The analysis is based on observational, survey-derived data aggregated at the location level, so associations do not imply causation and may be influenced by unmeasured confounders.

### 4.6. Discussion

The results of this study reveal several significant benefits for the healthcare domain.

**Enhanced Diagnostic Capabilities.** Advanced models demonstrated substantial improvements in early detection accuracy, evidenced by elevated R^2^ scores across testing scenarios, providing clinicians with more reliable tools for early-stage diagnosis. Models exhibited consistent performance across diverse patient populations, suggesting robust generalizability in clinical settings.

**Advanced Data Processing Architecture.** The integration of Llama3, GPT-4o, and GPT-5 embeddings with traditional machine learning approaches created an efficient processing pipeline for complex medical data. The system demonstrated exceptional capability in converting unstructured clinical narratives into quantifiable diagnostic indicators.

**Clinical Implementation Potential.** The framework shows immediate applicability in clinical environments, with minimal infrastructure requirements and potential for broader healthcare applications beyond Alzheimer’s disease detection. In our experiments, language-model embeddings augmented with geolocation features consistently outperformed the same embeddings without spatial context, validating the hypothesis that geolocation provides complementary information to textual signals and strengthening the case for hybrid systems in AI-assisted diagnostics.

## 5. Conclusions

This research has demonstrated the potential of leveraging advanced NLP models, in particular GPT-5, for the early detection of Alzheimer’s disease from patient records. By integrating language-model embeddings with a Random Forest Regressor and including geolocation features, the DMV framework achieved substantial gains in predictive accuracy. The best-performing configuration was GPT-5 with geolocation (MSE = 14.0339, MAE = 2.3715, R^2^ = 0.9783), markedly improving over the baseline RF without geolocation (MSE = 39.1041, MAE = 4.3668, R^2^ = 0.9294).

Our analysis also showed that adding geolocation features reduces MSE by roughly 48.6% for the RF baseline and by 52.0-58.2% for the embedding-based models (Llama3-70B, GPT-4o, GPT-5), confirming that spatial context provides complementary information to textual embeddings and improves AD risk estimation.

The research contributes to the growing body of work exploring the application of NLP in healthcare, particularly in the early detection of neurodegenerative diseases like Alzheimer’s. The findings underscore the value of utilizing state-of-the-art language models in clinical settings, where timely and accurate diagnosis can significantly impact patient outcomes. By demonstrating the effectiveness of GPT-5 in this context, the study paves the way for further integration of high-performance embedding models into clinical workflows.

Moreover, the research highlights the potential for advanced NLP models to process unstructured medical text, offering a scalable and automated solution for early disease detection. This capability is particularly relevant in scenarios where manual review of patient records is impractical due to the sheer volume of data.

Building on the success of this research, future work will focus on expanding the dataset by collaborating with hospitals and healthcare institutions to obtain a more diverse and comprehensive collection of patient records. In this way, we can further develop a more holistic approach to Alzheimer’s detection that could be implemented in clinical practice to assist healthcare professionals in making timely and informed decisions.

### 5.1. Limitations

The study is limited by its reliance on static datasets that may not capture real-time changes in patient conditions or emerging risk factors. In addition, the computational resources required for the framework may pose scalability challenges for healthcare institutions with limited infrastructure.

### 5.2. Ethical Considerations and Privacy

The integration of geolocation data in medical AI systems raises essential ethical and privacy considerations that must be addressed prior to clinical deployment. Geolocation information is highly sensitive, potentially revealing patients’ daily routines, socioeconomic status, and lifestyle patterns. To ensure responsible use of this technology, we recommend implementing several safeguards.

First, all geolocation data should be aggregated at appropriate geographic levels, like ZIP code or census tract, rather than precise coordinates, to prevent individual identification. Second, differential privacy techniques should be applied to protect against re-identification attacks. Third, explicit informed consent must be obtained from patients, clearly explaining how their location data will be used and protected. Fourth, data retention policies should minimize storage duration, and patients should have the right to opt out or request data deletion.

Healthcare institutions implementing this framework must comply with relevant regulations, including HIPAA in the United States, and ensure that the benefits of improved diagnostic accuracy outweigh potential privacy risks. Regular privacy impact assessments and independent audits should be conducted to maintain public trust.

### 5.3. Clinical Implementation Roadmap

The translation of our DMV framework into clinical practice requires a phased implementation approach. Phase 1 involves pilot testing in selected healthcare facilities with a robust IT infrastructure, integrating the system with existing Electronic Health Record (EHR) systems through FHIR-compliant APIs. During this phase, the model would operate in parallel with traditional assessment methods, allowing for validation against clinical outcomes.

Phase 2 focuses on refinement based on real-world performance metrics and clinician feedback. This includes adapting the interface for different clinical workflows, training healthcare staff, and establishing clear protocols for interpreting and acting on risk scores. Continuous monitoring systems would track model drift, ensuring that predictions remain accurate as patient populations and clinical practices evolve.

Phase 3 involves broader deployment with established feedback loops for continuous improvement. Post-deployment monitoring would include regular audits for bias, performance degradation, and unintended consequences. Collaboration with healthcare providers would ensure that the tool augments rather than replaces clinical judgment, with clear guidelines on when human oversight is essential.

## Figures and Tables

**Table 1:** Sample Questions and Risk Scores.

Questions	Risk Score
Percentage of older adults who are experiencing frequent mental distress	9.0
Mean number of days with activity limitations in the past month	6.1
Percentage of older adults currently not providing care who expect to provide care for someone with health problems in the next two years	14.5
Percentage of older adults who are currently obese, with a body mass index (BMI) of 30 or more	69.4
Percentage of older adults who self-reported that their health is “good”, “very good”, or “excellent”	72.9

**Table 2:** All Eleven Categorical Features in Text Type.

Categorical Features	Description	Example
datasource	Data Source	BRFSS
class	Class description	Mental Health
question	Question	Percentage of older adults who are experiencing frequent mental distress
topic	Topic description	Frequent mental distress
Data_Value_Type	The data value type	Age-adjusted prevalence or crude prevalence
stratification1	Identifier 1	50 years old
stratificationcategory1	Identification 1 value	Age Group, Sex, or Race/Ethnicity
stratification2	Identifier 2	ASN (Asian)
stratificationcategory2	Identification 2 value	Sex or Race/Ethnicity
locationabbr	Location Abbreviation	PA
locationdesc	Location Description	Pennsylvania

**Table 3:** All Five Numerical Features in Number Type.

Numerical Features	Description	Example
yearstart	Year Start	2022
latitude	Latitude	−77.86070029
yearend	Year End	2022
longitude	Longitude	40.79373015
data_value	Answer to question	48

**Table 4:** Metric Comparison (with Geolocation).

Metric	Baseline (RF)	Llama3-70B	GPT-4o	GPT-5
MSE	20.1164	16.6892	16.6118	**14.0339**
MAE	2.9352	2.8776	2.8833	**2.3715**
R-Square	0.9588	0.9695	0.9696	**0.9783**

**Note:** Bold values indicate the best performance for each metric.

**Table 5:** R^2^ Mean and R^2^ Std per Model (10-fold CV).

Models	R^2^ Mean	R^2^ Std
Baseline RF (w/ Geo)	0.9561	0.0006
Llama3-70B (w/ Geo)	0.9658	0.0007
GPT-4o (w/ Geo)	0.9668	0.0007
GPT-5 (w/ Geo)	**0.9765**	0.0007
Baseline RF (no Geo)	0.9276	0.0013
Llama3-70B (no Geo)	0.9446	0.0013
GPT-4o (no Geo)	0.9399	0.0015
GPT-5 (no Geo)	0.9462	0.0015

**Note:** Bold values indicate the best performance for each metric.

**Table 6:** MSE Comparison with/without Geolocation.

Models	w/Geo	w/o Geo	Relative Reduction
Baseline (RF)	20.1164	39.1041	48.56%
Llama3-70B	16.6892	34.7734	52.02%
GPT-4o	16.6118	34.8587	52.36%
GPT-5	**14.0339**	**33.5717**	**58.20%**

**Note:** Bold values indicate the best performance for each metric.

**Table 7:** MAE Comparison with/without Geolocation.

Models	w/Geo	w/o Geo	Relative Reduction
Baseline (RF)	2.9352	4.3668	32.77%
Llama3-70B	2.8776	4.0814	29.51%
GPT-4o	2.8833	4.2782	32.61%
GPT-5	**2.3715**	**4.0139**	**40.93%**

**Note:** Bold values indicate the best performance for each metric.

**Table 8:** R^2^ Comparison with/without Geolocation.

Models	w/Geo	w/o Geo	Relative Change
Baseline (RF)	0.9588	0.9294	+3.16%
Llama3-70B	0.9695	0.9461	+2.47%
GPT-4o	0.9696	0.9413	+3.01%
GPT-5	**0.9783**	**0.9480**	**+3.20%**

**Note:** Bold values indicate the best performance for each metric.

**Table 9:** Paired *t*-test results comparing per-fold MSE with and without geolocation.

Models	MSE (w/Geo)	MSE (w/o Geo)	Improvement	*t*-Statistic	*p*-Value
Baseline RF	20.1164	39.1041	48.56%	−50.1988	<0.001
Llama3-70B	19.6892	34.7734	43.38%	−44.9429	<0.001
GPT-4o	19.6118	37.8587	48.20%	−49.7768	<0.001
GPT-5	**14.0339**	**33.5717**	**58.20%**	−68.0977	<0.001

**Note:** Bold values indicate the best performance for each metric.

**Table 10:** Wilcoxon signed-rank test statistics for per-fold MSE differences.

Model	Wilcoxon Statistic	*p*-Value
Baseline RF	78,368,848	<0.001
Llama3-70B	82,601,294	<0.001
GPT-4o	78,451,943	<0.001
GPT-5	60,386,461	<0.001

## Data Availability

The data presented in this study are fully publicly available.
